# The Use and Abuse of Drugs in Tuberculosis

**Published:** 1908-08

**Authors:** William M. Jones

**Affiliations:** High Point, N. C.


### THE USE AND ABUSE OF DRUGS IN TUBERCULOSIS.*

BY WILLIAM M. JONES, M. D., HIGH POINT, N. C.

To what and where are we drifting in our therapeutics regarding tuberculosis and particularly that form known as Phthisis?

Since the beginning of Medical History, tuberculosis has been attacked from every known point, and by all kinds of men, with as many different varieties of treatment as was possible for the brain to conceive of. Why is it that we of today have not profited more by success or failure of our professors? Is it because of the fact that we have lost sight of the minor considerations in our insane endeavor to obtain a specific? We know that this disease has been successfully treated by men of the past, and that our results of today are but little more encouraging, than those of a hundred years ago.

By an examination of the history of the past few years, we find that the favorite of today, has invariably been the outcast of tomorrow. This has caused no small amount of distrust for the use of drugs, and we stand ready to discard them from our armamentum, and to rely entirely, on climatic, dietetic, and hygienic measures. That drugs are of value no one can deny,

Read before N. C. Medical Association, June 15, 16 and 17th 1908.

injury, the fatility of treatment applied to the seat of pain is although there may be differences of opinion, as to the position they occupy, when compared with other methods of treatment.

The narrow scope of this paper will not permit me to enter into any discussion other than the internal administration of drugs, and in this I shall not attempt to discuss their physiological action, or to mention more than a few of the more frequently employed, and confining my remarks in the main to such data as I have gained by observation.

Post-Mortem statistics present conclusive evidence of the curability of this disease, when we remember that there is at least as great a per cent, as 90 per cent. to 98 per cent. of all persons who reach the age of 35 years, who show healed tubercular lesions, we must infer that drugs have been instrumental to an extent in this restoration.

Caswell in 1836 said: "Pathological anatomy has never afforded stronger evidence of the curability of a disease than in the case of Phthisis."

Drugs used in tuberculosis are supposed to act in one of two ways; directly on the Tuberculae Bacillae, causing their destruction, or prohibiting their growth and proliferation; indirectly by the increase of weight and consequent increase in the strength and resistance of the patient. In regard to the destruction of the T. B., there is no drug or combination, of such strength, that will not at the same time destroy the surrounding tissues, though some may have the power to lessen their growth and proliferation.

Tuberculosis is seldom such, pure and simple. By this I mean to convey the idea that tuberculosis per se, is rarely to be found as the only pathological condition that is affecting the patient; even in early incipient cases there is often a mixed infection, and a slight leucocytosis with anemia.

When we use the term Abused, we do with the intention of conveying the idea, that it is contraindicated, whether from a physiological standpoint, or one of idiosyncrasy, or on account of some complication that is affecteing the patient, and thereby contradicts its administration.

The first that we shall consider is Cod Liver Oil, this is a fixt oil obtained from the fresh livers of the Gadus Morrhea, or other forms of Cod-Fish. This drug, or food as it is sometimes called, has to the mind of the laity become almost synomyous with tuberculosis and to such an extent that whenever a patient is taking cod-liver oil the inference is that he has consumption.

In the administration of this drug, it will be found advisable to begin with a small dose and gradually increase, using a few drops of Ether to assist in the digestion, and a small amount of oil. Eucalyptos may be added to disguise the taste. By this means patients will be enabled to consume larger amounts and for a longer time without the usual amount of digestive disturbances, that so frequently accompany its administration. The most that can be said of it, is that it is an easily assimilable fat, and as such causes an increase in the weight and strength of the patient, and thereby an increase in his resistance. We are aware of the fact that the market is simply deluged with patent and proprietary preparations, purporting to contain from 40 to 50 per cent. of oil, when in reality they seldom contain more than 8 to 10 per cent. and this not always cod-liver oil, but some substitute. Many of these preparations contain alcohol, and are therefore stimulants and not nutrients. Whenever this drug can be used without derangement of digestion, or other inconvenience to the patient, it is of advantage; on the other hand, when the opposite effects are produced, it is of very decided disadvantage, and its administration should be immediately discontinued.

The same may be said of other oils as of cod-liver oil, and cotton seed oil is preferred by many on account of the fact that it may be administered for a long time, and in larger doses, without so much derangement of digestion, and little inconvenience as regards taste, and on account of the fact that it may be administered during the hot months of summer.

Creosote, like cod-liver oil has become so fixed in the minds of the public, that they think that its only therapeutic use is in tuberculosis. The creosote that is usually found on the market is an impure phenol; and it is only the pure beechwood that should be used for internal administration. The employment of creosote in tuberculosis was based on the statement of Guttman: "That T. B. were destroyed by blood containing one of creosote to 2,000 and that one-half of that proportion would arrest their development." This was only theoretical, for we know that what good effects are derived from creosote are due to the formation of soluble compounds between the remedy and the toxic albumenous by-products of the T. B. which are eliminated by the blood.

The carbonate of guiacol is preferred by some clinicians, as it is the principle ingredient and of more definite c mpoistion. Creosote was first advanced as a remedy in tuberculosis by Reichenbach in 1833, and revived by Gimbert and Bouchard in 1877, since which time it has been used by men of reputation and ability, who have reported so favorably and in so many instances, that there is at least strong prima faci evidence in facor of creosote and its derivatives. But we must not lose sight of the fact that it is often the cause of digestive derangements, and when ever this is the case, its use should be immediately discontinued. One specialist has said as regards the digestive derangements: "In the majority of the cases sent to me, the digestive troubles, may be attributed to excessive doses of creosote, which had been administered by the family physician." It would be an act of presumptuousness, for me to attempt to discuss the great disadvantage to which a physician is placed in his endeavor to treat a case in which the stomach is weak and non-retentive to most of his remedies. Nevertheless this is a state of affairs or condition that we are often compelled to face, and one that will tax our patience, skill, and ingenuity to the utmost.

Ichthyol (Ammonia-Ichtryol-Sulphate) this though a comparatively new preparation, being first brought to the attention of the profession, as a remedy for tuberculosis in 1894 by Cohn and Scarpa, since which time it has been extensively employed and the results have exceeded the hopes and expectations of the most sanguinous.

It retards the disintegration of albuminoid substances and favors their formation and asismilation, increases peristalsis, and has a laxative action on the whole intestinal mucus. It is excreted by the feces nd urine, but not directly, as it is first absorbed and then excreted, this being verified by the fact that from seven to ten hours are consumed in its elimination. It causes an increase in body weight, lessens cough, and alters expectoration from purilant to mucoid, stimulates the digestion, thereby causing an increase in the appetite, and lessens nightsweats. Though not very pleasant to the taste it is seldom the cause of nausea. It may be administered in pill, capsule, or emulsion, and should in all cases be well diluted with milk or water, preferably immediately after meals. Of all drugs used in tuberculosis, I consider this far the superior, not alone from my own observation, but from the reports of well known physicians, and those of reputable institutions. It has been said, "that the only objection to its use was the cost."

A few words regarding the use and abuse of drugs when administered for special symptoms or complications.

It is in the management of the cough that drugs are more abused than in any other complication affecting a case of tuberculosis. The patient complains that he is annoyed to such an extent that he cannot sleep, on account of the harassing cough, and seeks relief from this distressing condition. In the majority of instances, he is given some mixture containing opium, or some preparation of a sedative and hypnotic character, and possibly containing a little potassium iodide, in a menstrum of syrup, that will mask the symptoms and aggravate the condition. The cough may have been due to some cardiac derangement, the tereminal fillaments of the vagus may have been irritated, and elongated uvula, inflammation of the eustation tube from middle-ear disease, laryngitis, post-nasal polip, etc. In the event that you are unable to find the direct exciting cause of the cough, and it is of such character, that relief is imperative, a little Heroin will in the majority of cases be all that is necessary.

Antipyretics have no place in the treatment of tuberculosis, as they only mask a symptom and do not affect a cause, with consequent depression of the heart and that of the general system. Rest absolute of mind and body will bring about all the reduction that is neecssary, and in the event that it does not reduce it, antipyretics are not called for.

Hemorrhage.—The drug most often used, and one that some of the recent text-books recommend is Ergot. Ergot is of value in hemorrhage of any organ with the exception of the lungs, here it is as surely contraindicated, and more so than that of any drug that I can at present call to mind. The reason is that ergot is a stimulant to the vasor-motor centre, causing a contraction of the small arteries and arteroles, and by this action lessening the escape of blood, but at the same time causing an increase in arterial tention and raising the blood pressure. The lungs being separate and distinct in their minute histological anatomy, as to circulation, accounts for the contraindication in the administration of ergot. The terminal branches of the pulmonary artery do not anastamose as do the terminal branches of other arteries, and the walls of the pulmonary vessels are much thinner and consequently weaker than others, and as a natural sequence when ever the blood pressure of the entire anatomy is raised, these weaker vessels are the first to give way and rupture under the increase, and the condition is thereby aggravated. Rest combined with small doses of atropine will generally stop the flow in a short time.

